# Prognostic Value of Circulating Microvesicle Subpopulations in Ischemic Stroke and TIA

**DOI:** 10.1007/s12975-019-00777-w

**Published:** 2020-01-25

**Authors:** Annika Lundström, Fariborz Mobarrez, Elisabeth Rooth, Charlotte Thålin, Magnus von Arbin, Peter Henriksson, Bruna Gigante, Ann-Charlotte Laska, Håkan Wallén

**Affiliations:** 1 Division of Internal Medicine, Department of Clinical Sciences, Karolinska Institutet, Danderyd University Hospital, SE-182 88 Stockholm, Sweden; 2 Department of Medical Sciences, Division of Cancer Pharmacology and Computational Medicine, Uppsala University, Uppsala, Sweden; 3 Division of Cardiovascular Medicine, Department of Clinical Sciences, Karolinska Institutet, Danderyd University Hospital, Stockholm, Sweden; 4 Division of Cardiovascular Medicine, Department Medicine, Karolinska Institutet, Stockholm, Sweden

**Keywords:** Ischemic stroke, TIA, Microvesicles, Platelet, Phosphatidylserine

## Abstract

Platelet microvesicles (PMV) have previously been found elevated in acute ischemic stroke (IS) and could be biomarkers for risk of recurrence. PMV surface antigens such as P-selectin and phosphatidylserine (PS) reflect platelet activation and procoagulance. Tissue factor-positive microvesicles (TF^+^MV) are considered procoagulant, in particular if co-expressing PS. We enumerated MV subpopulations with these surface antigens in a cohort of 211 patients with primarily non-cardioembolic IS or transient ischemic attack (TIA) and investigated their association with long-term outcome. MV concentrations were determined by flow cytometry in the acute and convalescent phase. Primary outcome was a composite of fatal and non-fatal recurrent IS or myocardial infarction. Secondary outcomes were recurrent IS and all-cause mortality. Outcome events were obtained from Swedish registers during a follow-up of 1100 patient years. Concentrations of PS-positive and PS-negative MV populations were elevated in patients compared with healthy controls in both the acute and convalescent phase. PS^+^TF^+^PMV displayed pronounced elevations, median fold change 77 in the acute phase (*p* < 0.0001) but were not associated with outcome, neither were PS^+^P-selectin^+^PMV. The only subpopulation positively associated with primary outcome was PS^−^TF^+^PMV, with adjusted hazard ratio of 1.86 (1.04–3.31, *p* = 0.036) by Cox regression. Unexpectedly, several MV subpopulations tended to be associated with reduced risk of poor long-term outcome. Our results suggest that PS^+^TF^+^PMV may be a promising marker for cerebral ischemia, and that the in vivo generation of PS^−^MV after IS/TIA warrants further study. Future MV studies should ideally enumerate PS^+^ and PS^−^MV subpopulations separately.

**Electronic supplementary material:**

The online version of this article (10.1007/s12975-019-00777-w) contains supplementary material, which is available to authorized users.

## Introduction

Ischemic stroke (IS) is one of the most common cardiovascular diseases and a major cause of morbidity and mortality globally [1]. Platelet function has a central role in IS; platelet activation is enhanced and remains elevated several months after an acute event [2–4]. Platelet activation causes release of platelet-derived microvesicles (PMV) which are 0.1–1.0 μm vesicles that bud off from the plasma membrane and can be detected in peripheral blood as markers of platelet activation. The procoagulant function of PMV was noted already at their discovery [5] and is attributed to exposure of phosphatidylserine (PS) and binding of coagulation factors [6–9]. More recently, the role of PMV in atherosclerosis/vascular inflammation and also in tissue regeneration has been recognized [10, 11]. Notably, PMV are heterogenous, with morphology, surface antigens, and content depending on platelet agonist(s) and pathological circumstances [12–14].

Tissue factor-positive microvesicles (TF^+^MV) are of interest for thrombotic diseases such as IS. TF, a main initiator of coagulation, was originally thought to reside exclusively on cells of the “hemostatic envelope” [15]. Ground-breaking work around the turn of the century demonstrated the existence of circulating MV-expressing TF, which were found to incorporate into thrombi and supported fibrin formation [16, 17]. Coexpression of PS on TF^+^MV is thought to increase procoagulance further [18, 19]. It has been postulated that primarily monocytes release TF^+^MV into the circulation [18, 20]. We and others have previously detected circulating TF^+^PMV [21, 22]. This finding is controversial as platelets are generally not thought to express TF [20]. However, two in vitro studies show that leukocyte TF can be transferred to platelets [23, 24]. Circulating TF^+^MV may thus reflect monocyte activation, and circulating TF^+^PMV may reflect platelet and leukocyte activation, or their interactions. Cell-cell interactions and the crosstalk between the hemostatic and innate immune systems at the vessel wall are increasingly recognized as critical for both thrombosis and inflammation; the terms “immunothrombosis” and “thromboinflammation” have been proposed [25, 26]. Thromboinflammatory processes, including generation of MV, are likely activated in ischemic cerebral vasculature and ischemia-reperfusion injury after stroke, especially as brain injury in stroke causes peripheral immune responses characterized by both activation and suppression [27]. PMV and TF^+^MV are thus interesting candidate biomarkers for IS. Previously, levels of PMV, TF^+^MV, and MV with TF activity were found to be elevated in IS [28–31], but as yet there is a lack of prospective studies, establishing prognostic value.

Importantly, recent findings suggest that MV biology is more complex than previously recognized. Flaumenhaft et al. showed that megakaryocytes constitutively release MV which may be counted as PMV when detected in peripheral circulation; these MV express PS but *not* P-selectin [32]. It has therefore been proposed that P-selectin-positive PMV are more suitable markers of platelet activation than PMV overall [21, 33]. Also, while PS expression is considered intimately associated with MV release [34, 35], Arraud et al. showed by cryo-transmission electron microscopy that about half of PMV in healthy persons do not express PS as detected by annexin V [36]. Notably, little is known about such PS^−^MV and their role in health or disease.

The aim of this study was to perform subtyping of PMV and TF^+^MV in patients with IS/TIA and to determine associations between MV populations and long-term outcome. Based on previous work on acute coronary syndrome (ACS), we expected the majority of MV to be PMV and TF^+^MV to be rare [21]. We hypothesized that the strongest association to poor outcome would be found for (a) PS^+^P-selectin^+^PMV, reflecting “true” platelet activation without megakaryocyte contribution, and (b) PS^+^TF^+^MV with or without co-expression of platelet marker due to high procoagulant potential. With respect to PS-negative populations and their association with long-term risk, the study was considered hypothesis generating as this has not been previously studied in detail.

## Materials and Methods

### Study Population and Evaluation of Outcome

The study PROPPSTOPP recruited 249 patients with minor IS or TIA from two stroke units in Stockholm, Sweden (Danderyd Hospital and Södersjukhuset) between 2007 and 2009. The original study had two objectives: (1) to screen for occult atrial fibrillation (AF) (completed [37]) and (2) to measure MV and variables of hemostasis [38]. Samples were collected in the acute/subacute phase of the index event, as well as in the convalescent phase 1 month later. Inclusion criteria were IS or TIA within 14 days of inclusion and age above 65 years (initially the age limit was > 45 years, but it was raised to obtain a more representative stroke cohort). Exclusion criteria were AF (known or present on admission ECG) and inability to perform screening with a handheld ECG device after discharge. In total, 38 patients were excluded before follow-up, due to incorrect diagnosis, patient request, or complete lack of blood samples, resulting in a cohort of 211 patients. MV analyses were performed for 199 patients in the acute phase and for 189 patients in the convalescent phase. Patients received secondary prevention for IS/TIA and treatment of risk factors at the discretion of the responsible physician.

Primary outcome was a composite of fatal or non-fatal recurrent IS and acute myocardial infarction (AMI). Secondary outcomes were recurrent IS and all-cause mortality respectively. Events and dates of events were retrieved from the Swedish Register of in-patient care and the Register for cause of death and were verified against hospital records. Patients were followed up until 31 Dec 2014 and censored after a first event with respect to the outcome evaluated.

For comparison, 53 age- and sex-matched healthy controls were recruited. They were not treated for hypertension or hyperlipidemia and received no antithrombotic, oral corticosteroid, or SSRI medication. Mild hypertension (< 150 mmHg systolic blood pressure without treatment), mild to moderate hyperlipidemia (total cholesterol < 8 mmol l^−1^), limited chronic kidney disease (plasma creatinine < 140 μmol l^−1^), well-substituted thyroid dysfunction, and successfully treated cancer more than 2 years before inclusion was allowed.

### Clinical and Routine Laboratory Evaluation

Medical history, clinical/anthropometric measures, and routine blood samples were taken at inclusion. Stroke severity was estimated by National Institutes of Health Stroke Scale (NIHSS). Routine blood/plasma analyses were performed by the Laboratory of Clinical Chemistry, Karolinska University Hospital, Stockholm, Sweden. Estimated glomerular filtration rate (eGFR) was calculated from plasma creatinine by the Modification of Diet in Renal Disease (MDRD) formula.

### Blood Sampling and Plasma Preparation

Blood was drawn from an antecubital vein, with no or minimal stasis and after rest for at least 10 min in recumbent position. For healthy controls, patients in the convalescent phase, and most patients in the acute phase (56%), blood sampling was performed in the morning after an overnight fast. For the remainder of patients (44%), acute phase blood sampling was performed in a non-fasting state due to short time between inclusion and patient discharge, taking care to avoid blood sampling after a meal. Platelet-poor plasma (PPP) was prepared immediately from citrated whole blood by centrifugation at 2000×*g* for 20 min in room temperature (RT) and was aliquoted and frozen at − 80 °C until analysis. Blood samples were taken median 3 days (interquartile range (IQR), 2–5 days) after symptoms in the acute phase and 36 days (IQR, 34–39 days) after symptoms in the convalescent phase.

### Microvesicle Analyses

The process for MV isolation and labeling has been described previously [21, 39]. In short, frozen PPP was thawed in waterbath (37 °C) for approximately 5 min and centrifuged at 2000×*g* for 20 min in RT. The supernatant (SN) was re-centrifuged at 13,000×*g* in RT for 2 min. Twenty microliters of the final SN was incubated for 20 min in the dark with 5 μl each of the appropriate fluorescent surface markers: phalloidin-Alexa 660 for actin (Invitrogen, Paisley, UK), lactadherin-FITC for PS (Haematologic Technologies, VT, USA), mouse antihuman CD41-PC7 for GPIIb of the platelet GPIIbIIIa receptor (Beckman Coulter Inc., CA, USA), mouse antihuman CD62P-APC for P-selectin (BD, NJ, USA), and mouse antihuman CD142-PE for TF (clone HTF-1, BD, NJ, USA) or corresponding non-specific isotype mouse IgG-antibodies as negative controls.

**Fig. 1:**
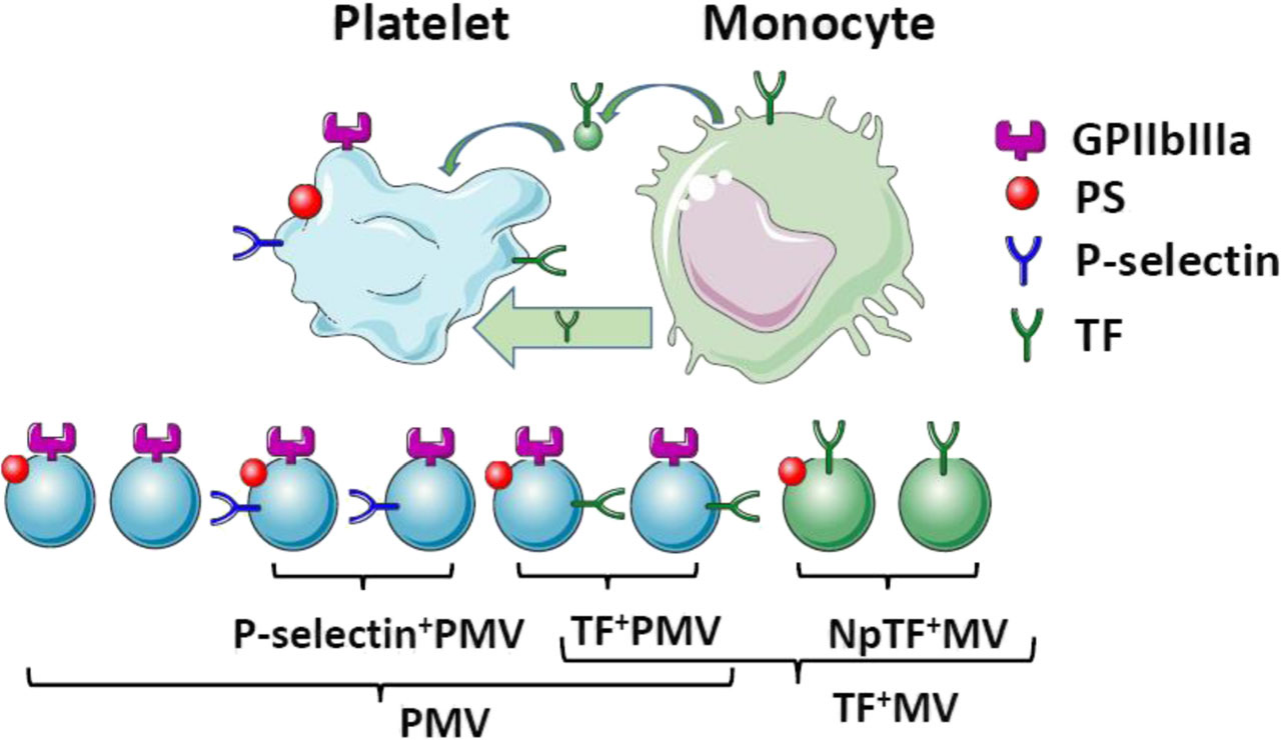
Schematic view of analyzed platelet microvesicles (PMV) and tissue factor (TF)-positive microvesicles (TF^+^MV) with their presumed cell of origin. Platelets express surface integrin GPIIbIIIa (dark purple) abundantly. Platelet activation causes exposure of phosphatidylserine (PS, red). Activated platelets release different PMV (light blue), positive or negative for PS. P-selectin-positive (dark blue) PMV (P-selectin^+^PMV) signal platelet activation including degranulation of α-granules. Primarily monocytes are thought to express TF (dark green). Monocyte TF may be transferred to platelets via uptake of monocyte MV (light green) or cell interactions (broad light green arrow). Subsequent platelet activation may lead to release of TF^+^PMV. Non-platelet TF^+^MV (npTF^+^MV, green) primarily from monocytes can also be released directly into the circulation. PMV lacking activation markers P-selectin and TF form a subset of PMV and were not enumerated separately. Sizes of cells, MV, and surface markers are non-proportional for clarity. The figure was created with images from Servier Medical Art, licensed under Creative Common Attribution 3.0 Unported License

Samples were analyzed in 2010 by a Beckman Coulter Gallios flow cytometer with established MV sensitivity and threshold set on forward scatter. The MV gate was determined by analyzing beads prior to samples (Megamix beads, 0.5, 0.9, and 3.0 μm; Biocytex, Marseille, France). MV events were defined as less than 0.9 μm in size as shown in Supplemental Fig. 1a. MV were labeled with lactadherin in order to optimize sensitivity for PS^+^MV [40]. The concentration of added lactadherin-FITC was 8 μg ml^−1^. The gating strategy to separate PS^+^ and PS^−^MV populations is shown in Supplemental Fig. 1b. Gating for enumeration of PMV subpopulations are shown in Supplemental Fig. 1c, d for PS-positive PMV; the same gating was used for PS-negative PMV. Gate settings were fixed for all analyses. Events were counted for 30 s at fixed flow rate (medium setting) and MV concentration calculated by dividing by flow volume as established by counting of beads of known concentration. MV enumeration by this method had inter- and intra-assay coefficients of variation less than 9% in a previous study by our group using a similar protocol [41]. Phalloidin-positive MV were used for sample quality control. Phalloidin binds actin of cell fragments which may be counted as MV. Good sample quality was defined as less than 10% phalloidin-positive events of total MV count. MV enumeration is presented as total number of MV with expression of the specified antigen(s), e.g., PS^+^PMV include all MV with surface markers PS and GPIIb including PS^+^P-selectin^+^PMV and PS^+^TF^+^PMV. An illustration of analyzed MV populations and their presumed cell of origin is shown in Fig. 1. TF^+^MV lacking the platelet marker GPIIb are denoted “non-platelet TF^+^MV” below.

### Statistical Methods

Normally distributed, continuous variables are presented as mean with standard deviations; non-normally distributed, continuous variables are presented as median with IQR. Differences between independent groups were analyzed by two-sided Student *t* test or Mann-Whitney *U* test as appropriate. Dependent variables were analyzed by paired Student *t* test or Wilcoxon test as appropriate. Correlations between independent variables were calculated by Spearman’s rank correlation coefficient, since several variables had non-normal distribution. Categorical variables are presented as numbers with proportions and were compared by *χ*^2^ test or Fisher’s exact test as appropriate.

The prospective analysis of outcome was performed in steps. In a first variable screening, patients with and without outcome events were compared by descriptive statistics and two-sided Student *t* test or Mann-Whitney *U* test as appropriate. Variables with *p* < 0.2 for difference were retained. In a second step, the cohort was divided by median split for the variable analyzed, with subsequent Kaplan-Meier analysis and log rank test for difference. Variables with *p* < 0.1 for difference were included in multivariate, forward stepwise Cox regression analysis adjusted for cardiovascular risk factors in order of importance. The number of variables in the final multivariate model was limited so as to have eight to ten patients with events per variable in order not to overfit the model. The number of analyzed patients with and without events for each test is presented in respective tables and figures.

*p* values < 0.05 were considered statistically significant in baseline analyses and in the final multivariate model. Data were analyzed by SPSS version 23 (IBM, IL, USA) and figures created by Prism (GraphPad Software Inc., CA, USA).

## Results

### Cohort Characteristics and Outcome

The clinical characteristics and laboratory data for the study cohort and healthy controls are presented in Table 1. Data from the Swedish Stroke Register are added for comparison where possible. The composite primary outcome occurred in 54 patients during a follow-up time of 1100 patient years. Forty-one patients had recurrent IS, twelve AMI (five of which were fatal), and one patient had both AMI and IS. Clinical characteristics of patients with and without primary outcome are included in Table 1. The secondary outcome recurrent IS occurred in 43 patients (including two who had AMI prior to recurrent IS). Thirty-one patients died. Five patients had a recurrent IS between acute and convalescent blood sampling. They were excluded from the prospective analysis of convalescent MV populations.

**Table 1:** Clinical and laboratory characteristics for the entire patient cohort, patients without and with primary outcome event (prim outcome), healthy controls and all Swedish stroke patients (data from the Swedish Stroke Register)

	Patient cohort	Patient without prim outcome	Patient with prim outcome	Healthy controls	Stroke register 2011
	*N* = 211	*N* = 157	*N* = 54	*N* = 53	*N* ≈ 25,000
Age at inclusion (years)	72.3 ± 8.2	71.5 ± 8.2	74.4 ± 7.9*	70.4 ± 7.8	75.9
Female (*n* (%))	89 (42%)	72 (46%)	17 (31%)	(42%)	48%
Medical history at inclusion					
Hypertension (*n* (%))	139 (66%)	101 (64%)	39 (72%)		61%
Diabetes (*n* (%))	32 (15%)	19 (12%)	13 (24%)*		24%
Active smoker (*n* (%))	38 (18%)	29 (19%)	8 (14%)		13%
Previous IS/TIA (*n* (%))	53 (25%)	35 (22%)	18 (33%)		37%
IHD (*n* (%))	38 (18%)	26 (17%)	13 (24%)		
TIA as index event (*n* (%))	72 (34%)	54 (34%)	17 (31%)		
NIHSS	0 (0–1)	0	0		
BMI at inclusion (kg m^−2^)	25.8 ± 3.8	25.9 ± 3.8	25.5 ± 3.9		
Hemoglobin (g l^−1^)	141 ± 13	141 ± 13	141 ± 15	137 ± 10	
Leukocyte count (× 10^9^ l^−1^)	6.9 (5.9–8.9)	6.9	6.9	5.0 (4.3–5.5)	
Platelet count (× 10^9^ l^−1^)	214 (181–262)	213	214	216 (184–235)	
Hs-CRP (mg l^−1^)	2.5 (0.9–6.4)	2.55	2.40	0.72 (0.27–1.14)	
P-glucose (mM)	6.4 (5.6–7.7)	6.3	6.5	5.4 (5.0–5.6)	
Creatinine (μM)	80 (70–93)	79	86*	70 (63–84)	
eGFR (ml min^−1^ 1.73m^−2^)	79 ± 20	80 ± 19	76 ± 22	89 ± 14	
LDL-cholesterol (mM)	3.2 ± 0.9	3.3 ± 1.0	3.0 ± 0.8*	3.6 ± 0.8	

*IS*, ischemic stroke; *TIA*, transient ischemic attack; *IHD*, ischemic heart disease; *NIHSS*, National Institute of Health Stroke Scale; *BMI*, body mass index; *eGFR*, estimated glomerular filtration rate

* signifies statistical probability *p* < 0.0001

Medication in the acute and convalescent phase as compared with the Swedish Stroke Register is shown in Supplementary Table 1. The vast majority of patients received antiplatelet treatment (96% in the acute phase and 95% in the convalescent phase) with aspirin being the most common treatment. A limited number of patients received anticoagulant treatment. Anticoagulation in the acute phase was generally low molecular weight heparin for prevention of venous thrombosis or due to multiple TIA. In the convalescent phase, 11 patients received warfarin, see Supplementary Table 1 for details.

Patients with primary outcome events were on average almost 3 years older and had higher prevalence of diabetes and higher plasma creatinine than patients without events, see Table 1. Unexpectedly, LDL-cholesterol in the acute phase was lower for patients with than without primary outcome, while there was no difference in statin treatment. LDL-cholesterol could thus not be used as a risk marker in this cohort. Based on Cox regression, cardiovascular risk factors had the following univariate associations to primary outcome (in order of importance): diabetes (HR, 2.2; *p* = 0.014), reduced kidney function with eGFR < 60 ml min^−1^ 1.73 m^−2^ (HR, 2.0; *p* = 0.028), age > 70 years (HR, 1.9; *p* = 0.038), and male sex (HR, 1.8; *p* = 0.054). Hypertension (*p* = 0.26) and smoking at inclusion (*p* = 0.52) were less clear risk factors in this cohort.

### Baseline MV Concentrations

MV isolation was of good quality with median 6.0% (IQR, 3–9%) of events being positive for phalloidin [39, 42]. The concentrations of MV populations are shown in Table 2. For both patients and controls, the majority of MV were PS^+^, and the majority were PMV for both PS^+^/PS^−^MV. Levels of total MV concentrations and PS^+^/PS^−^MV were moderately elevated in patients compared with healthy controls.

**Table 2:** Concentrations of MV populations for patients in the acute and convalescent phase as compared with healthy controls

	Patients acute phase	Patients conv phase	Healthy controls
	*N* = 199	*N* = 189	*N* = 53
Total MV	25,613 ± 3845^***^	22,886 ± 4281^***^	15,036 (12809–16,425)
PS^+^MV	20,948 ± 3774^***^	17,015 ± 3778^***^	12,716 (9988–13,607)
PS^+^PMV	15,688 ± 3214^***^	12,807 ± 2851^***^	9364 (7855–10,001)
PS^+^P-sel^+^PMV	7118 (5452–8337) ^***^	4244 (3326–5270) ^***^	1026 (803–1180)
PS^+^TF^+^PMV	1843 ± 500^***^	1169 ± 328^***^	24 (18–32)
Np PS^+^TF^+^MV	163 (98–218) ^***^	112 (67–152) ns	87 (74–156)
PS^−^MV	4520 (3210–5841) ^***^	6095 (4802–6654) ^***^	2703 (2113–3200)
PS^−^PMV	3191 (2331–4314) ^***^	4249 (3457–4998) ^***^	1159 ± 335
PS^−^P-sel^+^PMV	2208 (1619–2904) ^***^	2899 (2375–3445) ^***^	205 (158–259)
PS^−^TF^+^PMV	468 (367–579) ^***^	366 (294–458) ^***^	32 (18–44)
Np PS^−^TF^+^MV	125 (70–207) ^***^	104 (55–165) ^***^	33 (25–50)

Presented as mean ± standard deviation or median with interquartile range (counts μl^−1^)

*np*, non-platelet

***p < 0.0001 compared with healthy controls

#### Concentrations of PMV and P-Selectin-Positive PMV

**Fig. 2:**
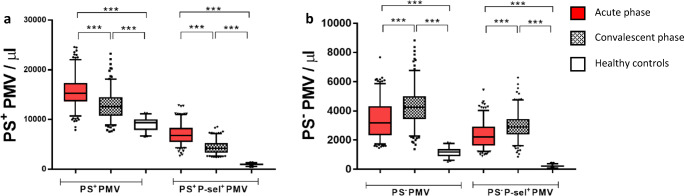
**a** Concentrations of PS^+^PMV and PS^+^P-selectin^+^PMV and **b** PS^−^PMV and PS^−^P-selectin^+^PMV. Red, patients in the acute phase; gray, patients in the convalescent phase; and white, healthy controls. ***p < 0.0001. Box, 25–75%; whiskers, 5–95%

Concentrations of PMV and P-selectin-positive PMV with respectively without PS are shown in Fig. 2, where Fig. 2a shows PS^+^ populations. PS^+^PMV were moderately elevated in the acute phase and decreased but remained significantly elevated compared with healthy controls in the convalescent phase. PS^+^P-selectin^+^PMV displayed pronounced elevations relative to healthy controls (fold change 7 in the acute phase), which decreased in convalescent phase. Figure 2b shows the corresponding PS^−^ populations. These display a different time profile, with *increasing* concentrations between the acute and convalescent phase.

#### TF^+^PMV Concentrations

**Fig. 3:**
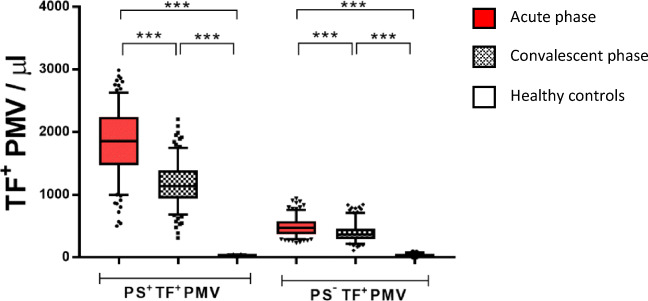
Concentrations of PS^+^TF^+^PMV and PS^−^TF^+^PMV. Red, patients in the acute phase; gray, patients in the convalescent phase; and white, healthy controls. ***p < 0.0001. Box, 25–75%; whiskers, 5–95%

Concentrations of TF^+^PMV populations are shown in Fig. 3. Patients displayed unexpectedly high concentrations of both PS^+^TF^+^PMV and PS^−^TF^+^PMV, median fold change 77 and 15 times (*p* < 0.0001) respectively in the acute phase compared with healthy controls. Levels decreased but remained substantially elevated in the convalescent phase. The proportion of PS^+^PMV expressing TF was median 12% in the acute phase, 9% in the convalescent phase compared with less than 3‰ for healthy controls. The corresponding figures for PS^−^PMV were 15 and 9% compared with 3% for healthy controls. Notably, for patients a clear majority of all TF^+^MV were TF^+^PMV (median 89% in the acute phase), while the opposite was the case for healthy controls (median 32%).

#### Non-platelet TF^+^MV Concentrations

**Fig. 4:**
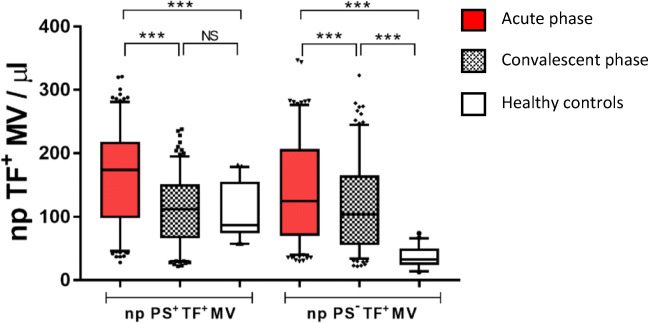
Concentrations of non-platelet (np) PS^+^TF^+^MV and PS^−^TF^+^MV. Red, patients in the acute phase; gray, patients in the convalescent phase; white, healthy controls. ***p < 0.0001; NS, non-significant. Box, 25–75%; whiskers, 5–95%

The concentrations of non-platelet TF^+^MV are shown in Fig. 4. In the acute phase, patients had a marginal but significant elevation of non-platelet PS^+^TF^+^MV compared with healthy controls, which normalized in the convalescent phase. Non-platelet PS^−^TF^+^MV displayed more pronounced elevations which were maintained in the convalescent phase.

#### Correlations Between MV Populations

As the generation mechanisms leading to circulating PS^−^MV are unknown, we analyzed correlations between PS^+^MV and PS^−^MV populations with otherwise the same surface markers. No substantial correlations were found for any of the populations, neither for patients in the acute or convalescent phase nor for healthy controls (Spearman’s correlation coefficient -0.25 < r < 0.25 for all, data not shown). For patients, marginal but significant *negative* correlations were found for PS^+^PMV and PS^−^PMV, respectively, PS^+^P-selectin^+^PMV and PS^−^P-selectin^+^PMV; Spearman’s correlation coefficient - (0.15–0.19) data not shown.

#### MV Concentrations Versus Clinical Factors

Comparing patients with IS and TIA as the index event, no significant differences were found in MV subpopulation concentrations measured in the acute phase. In the convalescent phase, patients with TIA had marginally lower PS^+^PMV than those with IS as index event (relative difference less than 10%); for other MV subpopulations there were no significant differences. There were no significant differences in MV concentrations depending on treatment (data not shown). There were no correlations between stroke severity measured by NIHSS points and MV concentrations.

### Prospective Analysis of MV Associations with Outcomes

#### Variable Screening and Univariate Analysis

**Fig. 5:**
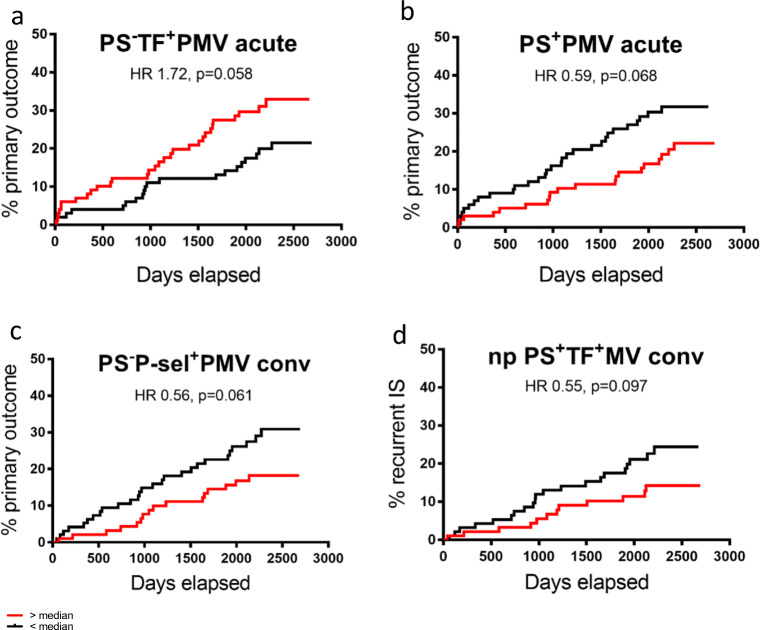
Univariate Kaplan-Meier curves for recurrent ischemic events versus respective MV concentrations above (red curves) and below median (black curves). **a** Primary outcome for patients with PS^−^TF^+^PMV above and below median in the acute phase. **b** Primary outcome for PS^+^PMV above and below median in the acute phase. **c** Primary outcome for patients with PS^−^P-sel^+^PMV above and below median in the convalescent phase. **d** Secondary outcome recurrent IS for patients with non-platelet PS^+^TF^+^MV above and below median in the convalescent phase

Univariate variable screening with descriptive statistics and Kaplan-Meier analysis based on median split was negative for a majority of the MV populations studied versus all outcomes. This included the primary candidate risk markers: PS^+^P-selectin^+^PMV, thought to reflect “true” platelet activation, and PS^+^TF^+^PMV, the dominant TF^+^MV population. Variable screening identified four MV populations with possible associations with outcomes; they are shown in Fig. 5. In the acute phase, PS^−^TF^+^PMV concentrations above median tended to be associated with an increased risk of primary outcome, see Fig. 5a. In contrast, PS^+^PMV above median in the acute phase appeared associated with an unexpected *reduced* risk for primary outcome, see Fig. 5b. In the convalescent phase, two populations showed possible associations with outcome PS^−^P-sel^+^PMV above median tended to be associated with a reduced risk of primary outcome, see Fig. 5c. Further, non-platelet PS^+^TF^+^MV tended to be associated with a reduced risk of the secondary outcome recurrent IS, see Fig. 5d. The number of events and patients analyzed for the respective populations are specified in Table 3.

**Table 3:** Result of univariate variable screening with Kaplan-Meier analysis based on median split with hazard ratio (HR) calculated by univariate Cox regression

	Primary outcome			Recurrent IS		
	HR	*N*	*p* (log rank)	HR	*N*	*p* (log rank)
Acute phase values						
PS^−^TF^+^PMV above median	1.72	50/199	0.058			
PS^+^PMV above median	0.59	50/199	0.068			
Convalescent phase values						
PS^−^P-sel^+^PMV above median	0.56	43/189	0.061			
PS^+^TF^+^GPIIb^−^MP above median				0.55	33/188	0.097

Variables with *p* < 0.1 for difference by log rank test for primary outcome and secondary outcome ischemic stroke (IS), respectively

*N*, number of cases of with event out of total number tested

None of the studied MV-populations was associated with all-cause mortality.

#### Acute-Phase Multivariate Analysis

A first step of the multivariate analysis was to determine whether acute-phase PS^−^TF^+^PMV and PS^+^PMV were independently associated with risk for primary outcome. Unexpectedly, correlation analysis showed that PS^−^TF^+^PMV and PS^+^PMV in the acute phase were moderately negatively correlated, Spearman’s correlation coefficient *r* = − 0.35 (*p* < 0.0001). These two variables were therefore not included in the same multivariate analysis.

A multivariate forward stepwise Cox regression model, adjusted for cardiovascular risk factors (in order of importance as established in “Cohort Characteristics and OutcomeS18”) was performed for PS^−^TF^+^PMV in the acute phase. The results are presented in Table 4. PS^−^TF^+^PMV was found to be a predictor of primary outcome after adjusting for diabetes, reduced kidney function, age, and sex, with HR 1.86 (1.04–3.31, *p* = 0.036).

**Table 4:** Multivariate Cox regression model for PS^−^TF^+^PMV in the acute phase versus primary outcome, adjusted for cardiovascular risk factors

Variable	HR	HR CI	*p* value
PS^−^TF^+^PMV above median	1.86	1.04–3.31	0.036
Diabetes at inclusion	2.10	1.06–4.12	0.032
eGFR < 60 ml min^−1^	2.03	1.03–4.00	0.042
Age at inclusion > 70 years	1.65	0.86–3.18	0.131
Male sex	2.27	1.22–4.24	0.010

*HR*, hazard ratio; *CI*, 95% confidence interval; *eGFR*, estimated glomerular filtration rate

The same model for PS^+^PMV in the acute phase resulted in the association between PS^+^PMV and primary outcome becoming increasingly non-significant after adjustment for cardiovascular risk factors (*p* = 0.212 in the final step of the model, data not shown).

#### Convalescent Phase Multivariate Analysis

In the multivariate analysis for convalescent phase variables, neither the association between PS^−^P-selectin^+^PMV and primary outcome nor the association between non-platelet PS^+^TF^+^MV and recurrent IS was significant after adjustment for cardiovascular risk factors.

## Discussion

In one of the first prospective studies to map and subtype PMV and TF^+^MV both positive and negative for phosphatidylserine (PS) in patients with IS/TIA, we show elevations of not only PS-positive but also PS-negative populations, which were maintained in the convalescent phase. In agreement with our hypothesis, PS^+^P-selectin^+^PMV showed more pronounced elevations compared with healthy controls than PS^+^PMV overall. Other results were unanticipated and contradicted original hypotheses. The main findings were:

1. Levels of TF^+^MV were unexpectedly high in patients and the vast majority co-expressed the platelet-specific marker GPIIb.
2. The primary biomarker candidates, PS^+^P-selectin^+^PMV, reflecting platelet activation, and PS^+^TF^+^PMV, the dominant TF^+^MV population, were not associated with outcome, despite having pronounced elevations in the acute and convalescent phase.
3. Only a few unexpected MV populations showed possible associations with outcome, two of which were PS negative and three of which tended to be associated with *reduced* risk.
4. PS^+^ and PS^−^ MV populations of the same type had few commonalities; they lacked correlations, had partly differing time profile after IS/TIA and differed in their associations with risk.

### Baseline TF^+^MV Concentrations

Levels of TF^+^MV co-expressing the platelet marker GPIIb (TF^+^PMV) were unexpectedly high in patients and exceedingly rare in healthy controls. In contrast, levels of non-platelet TF^+^MV were low, with limited elevations in patients compared with healthy controls. Thus, platelets contribute substantially to the generation of circulating TF^+^MV after IS/TIA. Furthermore, levels of TF^+^PMV were about one order of magnitude higher after IS/TIA compared with levels found for patients with ACS by our group using a similar MV protocol [21], i.e., TF^+^PMV generation appears substantially higher after cerebral compared with myocardial ischemia. Underlying mechanisms are unknown, but certain aspects should be mentioned. The specific central nervous system (CNS)-induced acute-phase response after IS/TIA or ischemia/reperfusion injury enhances chemokine expression in the liver, neutrophil mobilization, and activation of monocytes, Kupffer cells, and macrophages [27]. This may induce TF expression particularly in monocytic cells; TF could subsequently be taken up by platelets or transferred to platelets by cell-cell interactions. Another possible source of TF could be the ischemic/post-ischemic brain itself. Astrocytes in the brain are rich in TF, with particularly high levels close to perivascular spaces and subependymally [43]. High levels of circulating TF^+^PMV levels were previously found by our group in the acute phase of traumatic brain injury [44]. Astrocyte damage and/or activation after IS/TIA may result in leakage of TF to the blood, either by increased permeability of the blood-brain barrier or by clearance through the recently identified glymphatic and meningeal lymphatic systems [45]. Independent of underlying mechanisms, TF^+^PMV emerge as a promising biomarker for cerebral ischemia, as levels remain elevated for at least 1 month after IS/TIA.

### Associations Between MV Populations and Outcome

The prospective analysis disproved the original hypothesis that PS^+^P-selectin^+^PMV and/or PS^+^TF^+^PMV would be associated with poor outcome. Instead, these populations were neutral with respect to risk, despite showing pronounced elevations in both the acute and convalescent phase. The result illustrates the limitation of cross-sectional studies in evaluating MV as candidate biomarkers. In our previous study on ACS patients, PS^+^P-selectin^+^PMV nearly normalized and PS^+^TF^+^PMV decreased by half after 6 months [21]; this could be the case also after IS/TIA, which could contribute a lack of association with long-term outcome. It cannot be excluded that PS^+^P-selectin^+^PMV and PS^+^TF^+^PMV have associations with early recurrent events that the present study does not have the power to reveal. However, at present PS^+^P-selectin^+^PMV and PS^+^TF^+^PMV do not appear to be promising biomarkers of long-term risk after IS/TIA, despite theoretical appeal.

The MV populations identified as having possible associations with long-term outcome were unexpected, as were the risk relations found. The only population with increased risk was PS negative; PS^−^TF^+^PMV were significantly associated with primary outcome after adjustment for CVD risk factors. Since the generation mechanisms, target cells and clearance of PS^−^TF^+^PMV are unknown, it is unclear how this population mediates risk. Interestingly, a recent study on SLE patients showed PS^−^PMV to have stronger association to disease activity than PS^+^PMV populations [46], supporting the notion that PS^−^MV may have a role in pathophysiology. It would thus be of interest to characterize PS^−^TF^+^PMV further.

Three MV populations tended to be associated with *reduced* risks in univariate analysis: PS^+^PMV in the acute phase versus primary outcome, PS^−^P-selectin^+^PMV in the convalescent phase versus primary outcome and non-platelet PS^+^TF^+^MV in the convalescent phase versus recurrent IS. Adjustment for CVD risk factors resulted in non-significant HR for all. The limited number of events may have contributed to the lack of significance, as Kaplan-Meier curves showed consistent separation over time—significance may have been reached in a larger study. In general, MV are studied for their proinflammatory or procoagulant effects, and the concept that certain MV populations could be protective has so far received limited attention. However, PMV have been reported to mediate tissue repair and vascular remodeling, e.g., stimulating angiogenesis [10]. It was recently shown that topical application of PMV to the ischemic brain in a mouse model of ischemic stroke resulted in enhanced angiogenesis and neurogenesis, improving neurological outcome [47]. Such effects could explain the apparent positive effect observed.

### Comparison of PS^+^ and PS^−^ MV Populations

So far, PS^−^MV populations have received little attention, and many studies enumerate only PS^+^MV or total MV without a separate marker for PS. It is debated whether MV identified as PS negative completely lack PS or express PS at concentrations too low for detection with annexin V, the marker most commonly used [35, 40]. We have used the more sensitive PS-marker lactadherin [40], still finding a substantial proportion of MV to be PS negative. It has been proposed that PS-negative MV could be PS-positive at formation, with PS subsequently binding endogenous molecules such as lactadherin or β2-glycoprotein-1, which may mask PS epitopes [48, 49]. It has also been suggested the PS-negative MV would remain longer in the circulation, not being subjected to the same clearance mechanisms as PS-positive MV [50, 51]. High generation/high clearance of PS-positive MV combined with low generation/low clearance of PS-negative MV would be consistent both with PS-positive MV being the dominant generation mechanism and the relatively high number of circulating PS-negative MV found, e.g., by Arraud. Notably, our results provide little support for the notion that PS-positive and PS-negative MV subtypes are “two sides of the same coin.” If generation mechanisms were common, at least for healthy controls sampled under steady-state conditions, one would expect positive correlations between PS-positive and PS-negative MV of the same type. We found no important correlations for any of the groups tested, and the weak correlations in patients were negative. Further, the correlation between PS^+^PMV and PS^−^TF^+^PMV was clearly negative. For patients, the time profiles differed when comparing PS^+^/PS^−^ PMV respectively with P-selection^+^PMV. Also, associations between PS^+^/PS^−^ MV subpopulations and risk differed. Together, our results suggest important differences in circulating PS^+^MV and PS^−^MV subtypes, which may involve generation, binding to target cells and/or clearance. With respect to PS^+^/PS^−^ PMV, it is increasingly recognized that platelets adopt different phenotypes at activation, which are either positive or negative for PS [52, 53]. If PS^+^ and PS^−^ PMV are released from different and complementary platelet phenotypes, it would explain negative correlations. Further study of the nature and generation of PS^−^MV is warranted. From a biomarker perspective, our results suggest that PS^+^ and PS^−^ MV subpopulations should be considered separate entities and should be enumerated separately.

## Conclusions

In one of the larger clinical studies on MV after IS/TIA, TF^+^PMV were found strongly elevated in both the acute and convalescent phase and may be promising markers of cerebral ischemia. However, only the minor population PS-negative TF^+^PMV was positively associated with risk of new ischemic events. Several MV populations tended to be associated with reduced risk of poor outcome, suggesting possible protective effects. Our results illustrate that prospective studies of MV subtypes in relation to outcome has the potential to reveal pathological or indeed protective patterns of cell activation after IS/TIA.

### Limitations

Patients in the cohort differed in treatments that may influence MV generation, in particular, antithrombotic/antiplatelet treatment. Aspirin, the most common treatment, and warfarin are considered to have a limited influence on overall PMV levels; clopidogrel (given to a minority of patients) has been found to reduce PMV overall [54]. Aspirin (COX-1 inhibition) and clopidogrel (P2Y_12_ inhibition) also differ in their effect on platelet-leukocyte interactions and off-target pro- and anti-inflammatory effects [55, 56]. The study was not designed or powered to address these issues, but univariate analysis did not show significant differences in MV concentrations depending on treatment received.

Pre-analytical sample handling influences MV concentrations. We used PPP centrifuged at 2000×*g* for 20 min as the starting material, an established method at the time of blood sampling (2007–2009). Since 2013, ISTH recommends double centrifugation (2500×*g* for 15 min × 2) before freezing to exclude spurious PMV from in vitro platelet activation [57]. As we used phalloidin to confirm low levels of residual cell fragments [39], we expect the error due to centrifugation to be limited. The sensitivity of flow cytometry to small MV is another limitation; we would estimate the lower limit of detection to be in the range 250–300 μm, i.e., just above the median size found for spherical MV in healthy in platelet-free plasma [36]. While smaller MV may be missed, we expect limited detection of exosomes, which are not the topic of this study. Also, large MV may have stronger clinical relevance while large PMV had a stronger activating effect on neutrophils and endothelial cells in vitro than small in one study [58], and a recent study found TF^+^MV to be in the larger size range [59].

## Electronic Supplementary Material

ESM 1 (DOCX 14 kb)

ESM 2 (DOCX 14 kb)
