# Supplementary material for: Prognostic Value of Circulating Microvesicle Subpopulations in Ischemic Stroke and TIA
Source: Transl Stroke Res. 2020 Jan 25;11(4):708–19. doi: 10.1007/s12975-019-00777-w (PMC7340656; doi:10.1007/s12975-019-00777-w)
Supplement: Supplementary file 1 — (DOCX 14 kb) [file 12975_2019_777_MOESM1_ESM.docx]

Supplement table 1: Medication in the acute and convalescent phase as compared to Swedish Stroke Register 2011. Warfarin was given to 6 patients after diagnosis of atrial fibrillation in the screening program, 2 due to previous indication (other than AF), 1 due to atrial aneurysm and 2 based on strong clinical suspicion of cardiac embolus. (Two of these patients received also antiplatelet treatment.) ACEI=angiotensin converting enzyme inhibitor, ARB=angiotensin receptor II blocker.

|  | Patients acute  phase  N=211 | Patients convalescent  N=206 | Stroke register 2011  N≈25 000 |
| --- | --- | --- | --- |
| Antitrombotic treatment |  |  |  |
| -Aspirin, n (%) | 139 (66%) | 118 (57%) | 57% |
| -Aspirin+dipyridamole, n (%) | 25 (12%) | 36 (18%) |  |
| -Clopidogrel, n (%) | 16 (8%) | 32 (16%) | 36% |
| -Aspirin + clopidogrel, n (%) | 4 (2%) | 4 (2%) |  |
| -Warfarin, n (%) | 4 (2%) | 9 (4%) |  |
| -Other/combinations, n (%) | 18 (8%) | 2 (1%) |  |
| -None | 4 (2%) | 2 (1%) |  |
| Antihypertensive treatment |  |  |  |
| -ACEI, n (%) | 48 (23%) | 62 (30%) | 35% |
| -ARB, n (%) | 38 (18%) | 41 (20%) | 14% |
| -Calcium inhibitor, n (%) | 51 (24%) | 54 (26%) | 27% |
| -β-blocker, n (%) | 66 (31%) | 63 (31%) | 43% |
| -Diuretics, n (%) | 33 (16%) | 32 (16%) | 29% |
| Statin treatment, n (%) | 129 (61%) | 164 (80%) | 60% |
