# Supplementary material for: Prognostic Value of Circulating Microvesicle Subpopulations in Ischemic Stroke and TIA
Source: Transl Stroke Res. 2020 Jan 25;11(4):708–19. doi: 10.1007/s12975-019-00777-w (PMC7340656; doi:10.1007/s12975-019-00777-w)
Supplement: Supplementary file 2 — (DOCX 14 kb) [file 12975_2019_777_MOESM2_ESM.docx]

**Cover letter**

Dear professor Zhang, dear editors,

We are grateful for your preliminary positive response on our paper ‘Prognostic value of circulating microvesicle populations in ischemic stroke and TIA’, manuscript TRSR-D-19-00332.

We have now shortened and revised the manuscript according to reviewer comments, in particular the Discussion chapter. We also include a list of itemized responses to comments and questions from reviewer #1 and #3. (We could not find any comments from reviewer #2 in your mail or in attachments.) To clarify measured MV populations and their presumed origin we have included a figure (figure 1) but are off course happy to instead provide a table if you prefer. We hope these revisions will support your decision to publicize. As added information part of the results were presented in poster form at the ISTH SSC conference in Dublin July 2018 and as an oral presentation at the 3^rd^ Maastricht Concensus Conference on Thrombosis February 2019; the results were also included in my thesis and dissertation June 2018.

Hoping for your favorable response. Kind regards,

Annika Lundström MD PhD	Håkan Wallén MD Professor

Karolinska Institutet Danderyd Hospital	Karolinska Institutet Danderyd Hospital

Dept of Clinical Sciences	Dept of Clinical Sciences

Division of Internal Medicine	Division of Cardiovascular Medicine
